# The Interplay Between Rheumatoid Arthritis and Chronic Kidney Disease: From Mechanisms to Treatment

**DOI:** 10.3390/jcm15010108

**Published:** 2025-12-23

**Authors:** Kunihiro Ichinose

**Affiliations:** 1 Department of Rheumatology, Faculty of Medicine, Shimane University, 89-1 Enya-cho, Izumo 693-8501, Japan; kichinose@med.shimane-u.ac.jp; Tel.: +81-853-20-2196; Fax: +81-853-20-2194; 2 Integrated Kidney Research and Advance, Faculty of Medicine, Shimane University, 89-1 Enya-cho, Izumo 693-8501, Japan

**Keywords:** rheumatoid arthritis, chronic kidney disease, renal impairment, inflammation, biologics, TNF inhibitor, IL-6 inhibitor, JAK inhibitor, nephroprotection

## Abstract

Chronic kidney disease (CKD) is a frequent and clinically significant comorbidity in patients with rheumatoid arthritis (RA), with a reported prevalence ranging from 20% to 50% depending on the cohort and definition applied. The high burden of CKD in RA reflects the complex interplay between traditional risk factors (aging, hypertension, diabetes, and dyslipidemia) and RA-specific factors such as persistent systemic inflammation, immune complex deposition, and long-term exposure to nephrotoxic agents, including older DMARDs (gold, D-penicillamine) and calcineurin inhibitors. Histopathologically, RA-associated kidney involvement encompasses a broad spectrum of conditions, including mesangial proliferative glomerulonephritis, membranous nephropathy, AA amyloidosis, and drug-induced interstitial nephritis. Recent advances in RA therapy, particularly the widespread use of biologic DMARDs, have markedly reduced the incidence of AA amyloidosis and may exert indirect renoprotective effects through stringent inflammation control. However, targeted synthetic DMARDs such as Janus kinase (JAK) inhibitors require careful dose adjustment in CKD and heightened infection vigilance. CKD in RA is a strong predictor of cardiovascular events, serious infections, and all-cause mortality. Importantly, recent data indicate that even low-grade albuminuria below the traditional microalbuminuria threshold is associated with excess mortality in RA. Early detection through routine monitoring of eGFR and urinary albumin-to-creatinine ratio (uACR), combined with individualized pharmacologic adjustment and close collaboration with nephrologists, is essential for optimizing long-term outcomes. This review provides an updated synthesis of the epidemiology, pathophysiological mechanisms, therapeutic strategies, and prognostic implications of CKD in RA, with a particular focus on both Japanese and international evidence.

## 1. Introduction

Rheumatoid arthritis (RA) is a chronic autoimmune inflammatory disease that primarily affects the synovial joints; however, its systemic nature often leads to extra-articular manifestations, including renal involvement. Chronic kidney disease (CKD) has emerged as a clinically relevant comorbidity in patients with RA, contributing to increased morbidity, mortality, and therapeutic complexity. The prevalence of CKD among patients with RA is significantly higher than that in the general population, with estimates ranging from 20% to 50%, depending on the study design and definitions employed [1,2,3].

Several mechanisms have been proposed to explain this increased susceptibility, including traditional risk factors such as aging, hypertension, diabetes, and dyslipidemia, as well as RA-specific factors such as persistent systemic inflammation, long-term use of nephrotoxic agents such as nonsteroidal anti-inflammatory drugs (NSAIDs), and adverse effects of disease-modifying anti-rheumatic drugs (DMARDs) [1,4,5]. The histopathological findings in RA-associated nephropathy are diverse, ranging from mesangial proliferative glomerulonephritis and membranous nephropathy to AA amyloidosis and drug-induced tubulointerstitial nephritis [6,7,8,9].

Advances in RA management, particularly the widespread use of biological and targeted synthetic DMARDs, have improved disease control and may help mitigate renal complications in patients with RA. Interleukin-6 (IL-6) inhibitors have shown promise in preserving renal function, particularly in patients with AA amyloidosis [10,11,12]. Nevertheless, the presence of CKD complicates therapeutic decision-making and requires careful dose adjustment and interdisciplinary collaboration.

This review aimed to provide a comprehensive overview of the epidemiology, pathophysiology, clinical management strategies, and prognostic implications of CKD in patients with RA by synthesizing evidence from clinical studies and basic research across diverse populations.

## 2. Methods (Literature Search Strategy)

This narrative review was conducted based on a comprehensive literature search of PubMed and Embase databases. The search included articles published up to March 2025. The following key terms and their combinations were used: “rheumatoid arthritis”, “chronic kidney disease”, “renal impairment”, “albuminuria”, “biologic DMARDs”, “IL-6 inhibitor”, “TNF inhibitor”, “JAK inhibitor”, “SGLT2 inhibitor”, and “glomerulonephritis”.

Only English-language articles were included. Both observational and interventional studies, as well as relevant systematic reviews and meta-analyses, were considered. Case reports were included only when they provided mechanistic insights or addressed rare renal manifestations. Editorials and non-peer-reviewed articles were excluded. Reference lists of key publications were also manually screened to identify additional relevant studies.

## 3. Epidemiological Background

Chronic kidney disease (CKD) is increasingly recognized as a frequent and clinically significant comorbidity in patients with RA. Epidemiological studies have reported a wide range of CKD prevalence in RA, with rates varying from 20% to 50%, depending on the population and diagnostic criteria used [3]. For example, the French COMEDRA cohort reported that 8.8% of patients with RA had an estimated glomerular filtration rate (eGFR) below 60 mL/min/1.73 m^2^ and 9% had proteinuria [1]. In a Japanese retrospective cohort study of 1077 patients with RA, the baseline prevalence of CKD, defined as an eGFR <60 or proteinuria ≥1+, was 24.5%, and the cumulative incidence over 10 years was 60% [2].

The growing burden of CKD in patients with RA is partly attributable to the aging RA population and improved survival resulting from early diagnosis and better disease control. Indeed, the average age of patients with RA has been rising globally, and comorbid conditions such as hypertension and diabetes, which are key risk factors for CKD, are more prevalent in older adults. Long-term follow-up studies have shown that patients with RA are more likely to progress to CKD than age- and sex-matched non-RA controls. For instance, in a 20-year cohort study, approximately 25% of patients with RA developed an eGFR < 60, compared to 20% in non-RA individuals [13].

Several risk factors for the development and progression of CKD in patients with RA have been identified. A multicenter Japanese study demonstrated that advanced age (≥65 years; odds ratio [OR], 5.19), hypertension (OR, 3.05), diabetes mellitus (OR, 1.52), dyslipidemia (OR, 1.53), and higher cumulative glucocorticoid exposure (OR, 1.45) were independently associated with a reduced eGFR [4].

In addition, the frequent use of nonsteroidal anti-inflammatory drugs (NSAIDs) increases the risk of new-onset CKD by approximately 1.4-fold and may increase all-cause mortality [1]. Genetic susceptibility may further influence the risk of developing CKD. For example, the HLA-DRB1*DR6 allele is associated with a higher CKD prevalence in Japanese patients with RA [14].

A substantial proportion of patients with RA exhibit low-grade albuminuria, and values below the traditional microalbuminuria range may provide prognostic information for these patients. In the National Health and Nutritional Examination Surveys (NHANES) from 1999 to 2018, the uACR–mortality curve in RA showed an L-shaped threshold near ~6 mg/g, suggesting that “normal-high” albuminuria warrants attention [15]. Independent cohorts have also been associated with microalbuminuria (uACR 30–300 mg/g) and higher mortality [16]. These data support the incorporation of albuminuria, not just eGFR, into risk appraisal.

From a measurement standpoint, Kidney Disease: Improving Global Outcomes (KDIGO) 2024 recommends staging CKD according to eGFR categories (G1–G5) and albuminuria categories (A1–A3), emphasizing periodic screening in at-risk populations in which annual eGFR/uACR is reasonable, and more frequent testing is justified in patients with active disease, nephrotoxic analgesic use, or existing CKD [17,18,19]. Finally, the therapeutic era matters: the decline of AA amyloidosis with modern anti-cytokine strategies likely alters the renal epidemiology of RA; however, drug-related nephrotoxicity (e.g., chronic NSAID exposure) remains a modifiable contributor to the CKD burden [17,20].

In Japanese data, concomitant CKD independently increased the risk of hospitalized infections among patients with RA, and infections (including sepsis) were the leading reasons for Intensive Care Unit (ICU) admission in a Japanese ICU cohort of patients with RA, underscoring infection vulnerability when renal impairment coexists [21,22].

Together, these findings suggest that both traditional and RA-specific factors interact to increase the risk of CKD in patients with RA. In particular, older patients with RA have a higher prevalence of renal impairment than younger patients. However, recent evidence suggests that maintaining a low disease activity may attenuate the rate of eGFR decline. A large prospective study showed that patients with RA with sustained low disease activity had slower renal function loss than those with high disease activity, highlighting the importance of inflammation control in preserving renal outcomes [23]. Over the past two decades, improved survival in patients with RA due to earlier diagnosis and the adoption of treat-to-target strategies has paradoxically increased the clinical burden of age-related comorbidities, including CKD. In Japan, the average age of patients with RA has increased considerably, further amplifying the impact of age-associated renal risks.

## 4. Pathophysiology

Renal involvement in RA occurs through both inflammatory and drug-induced mechanisms. Systemic inflammation in RA leads to elevated circulating cytokines, such as tumor necrosis factor-alpha (TNF-α) and IL-6, which contribute to glomerular injury, vascular dysfunction, and interstitial fibrosis. IL-6 levels are markedly elevated in advanced CKD and play a key role in promoting proteinuria, glomerular permeability, and renal fibrosis [24]. Cytokine-induced disruption of the glomerular basement membrane (GBM) can cause podocyte injury and loss of charge selectivity, resulting in proteinuria [25,26,27]. Beyond serving as a marker of glomerular injury, proteinuria itself actively contributes to the progression of kidney damage. Excessive filtration of albumin leads to its uptake by proximal tubular epithelial cells via endocytic pathways, triggering cellular stress responses and the release of pro-inflammatory and pro-fibrotic mediators. Extracellular vesicles (EVs), including exosomes as a major subclass, have emerged as important mediators of intercellular communication in the kidney. Experimental and translational studies have demonstrated that albumin overload induces the release of these EVs from tubular epithelial cells. These vesicles carry cytokines, chemokines, and microRNAs that propagate local inflammation, activate interstitial fibroblasts, and promote tubulointerstitial fibrosis. Thus, albuminuria represents not only a consequence of glomerular injury but also a direct driver of tubular inflammation and chronic kidney disease progression [26,27].

In RA, chronic systemic inflammation contributes directly to glomerular and tubulointerstitial injuries. Activated T lymphocytes and macrophages infiltrate the glomeruli and release cytokines, such as IL-6, TNF-α, and interferon-γ. These mediators enhance the permeability of the glomerular basement membrane (GBM), leading to protein leakage and mesangial activation. Immune complexes containing rheumatoid factor or anti-citrullinated protein antibodies may deposit along the subepithelial or mesangial regions, triggering complement activation and subsequent podocyte injury.

**Figure 1:**
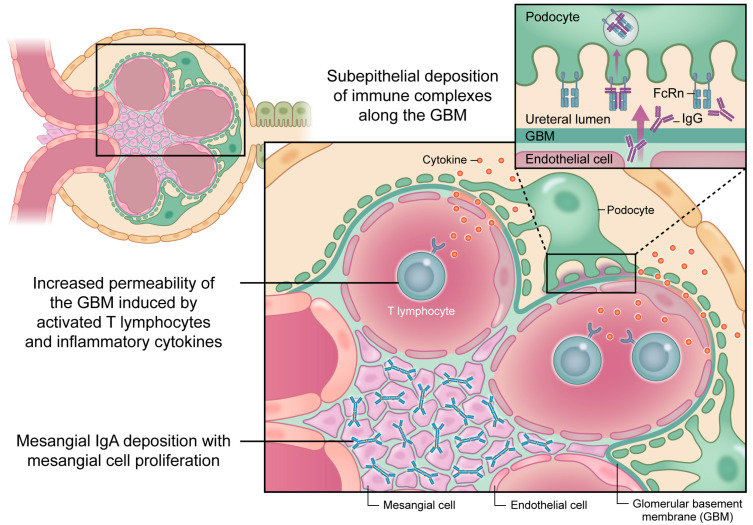
Immunological mechanisms of renal injury in rheumatoid arthritis. The diagram illustrates mesangial IgA deposition, mesangial proliferation, cytokine-mediated glomerular permeability, and subepithelial immune complex accumulation. Activated T cells and inflammatory cytokines damage the glomerular basement membrane (GBM) and podocytes, while FcRn-mediated IgG trafficking perpetuates proteinuria. Abbreviations: GBM, glomerular basement membrane; FcRn, neonatal Fc receptor; IgG, immunoglobulin G; IgA, immunoglobulin A.

Furthermore, podocytes can internalize IgG via the neonatal Fc receptor (FcRn), amplifying local inflammation and proteinuria. Collectively, these immunological events promote mesangial proliferation, endothelial activation, and progressive glomerular damage, as illustrated in Figure 1.

### 4.1. Immune-Complex-Mediated Renal Injury in Rheumatoid Arthritis

Renal involvement in rheumatoid arthritis (RA) has traditionally been attributed to immune-complex-mediated mechanisms. Circulating immune complexes containing rheumatoid factor and other autoantibodies may deposit within glomerular structures, leading to complement activation and inflammatory cell infiltration. Histopathological studies have demonstrated that membranous nephropathy and mesangial proliferative glomerulonephritis represent typical renal manifestations associated with these immune-mediated processes in RA [7,9]. Although the overall incidence of such classical immune-complex-mediated glomerular lesions has declined in the biologic era, these entities remain relevant, particularly in patients with long-standing or inadequately controlled disease [2,4].

### 4.2. AA Amyloidosis in the Biologic Era

AA amyloidosis has historically been a major cause of chronic kidney disease (CKD) and progression to end-stage renal disease in patients with poorly controlled RA. Persistent systemic inflammation results in sustained elevation of serum amyloid A, which promotes amyloid deposition within renal tissue and leads to progressive proteinuria and renal dysfunction [28]. The introduction and widespread use of biologic DMARDs, especially IL-6 inhibitors, have dramatically reduced the incidence and progression of AA amyloidosis in RA, as demonstrated in both Japanese and international cohorts [9,10,11]. Nevertheless, AA amyloidosis remains clinically relevant in selected patient populations, including those with delayed diagnosis, limited access to biologic therapy, or refractory inflammatory disease [12].

### 4.3. Drug-Induced Renal Injury and Nephrotoxicity

Drug-related nephrotoxicity continues to play an important role in the development and progression of CKD in patients with RA. Nonsteroidal anti-inflammatory drugs are well recognized to cause hemodynamic renal injury through inhibition of prostaglandin synthesis, leading to reduced renal perfusion and acute or chronic kidney dysfunction [29]. In addition, older disease-modifying anti-rheumatic drugs, such as gold salts and D-penicillamine, have been associated with immune-mediated interstitial nephritis and proteinuric renal disease [30]. Calcineurin inhibitors, including tacrolimus, may induce dose-dependent nephrotoxicity through sustained vasoconstriction, endothelial dysfunction, and promotion of renal fibrosis, particularly with long-term exposure [31]. These observations underscore the importance of careful drug selection and regular renal monitoring in patients with RA, especially those with pre-existing CKD.

### 4.4. Emerging Molecular Pathways Linking RA-Associated Inflammation and Renal Injury

Beyond classical immune-complex-mediated mechanisms, recent experimental studies have highlighted several molecular pathways that may bridge systemic inflammation and renal parenchymal injury in RA. Oxidative stress and activation of the renin–angiotensin system contribute to endothelial dysfunction, podocyte injury, and progressive glomerulosclerosis [32]. Inflammatory signaling through the IL-17–ERK/AKT pathway has been implicated in amplifying mesangial cell proliferation and tubular epithelial damage [33,34]. In addition, activation of the Wnt1/β-catenin pathway promotes fibroblast activation and renal interstitial fibrosis, thereby accelerating CKD progression [35].

At present, most supporting evidence for these pathways derives from experimental and preclinical studies, and their direct relevance to human RA-associated CKD requires validation through future translational and clinical investigations.

### 4.5. Summary of Mechanistic Pathways

**Figure 2:**
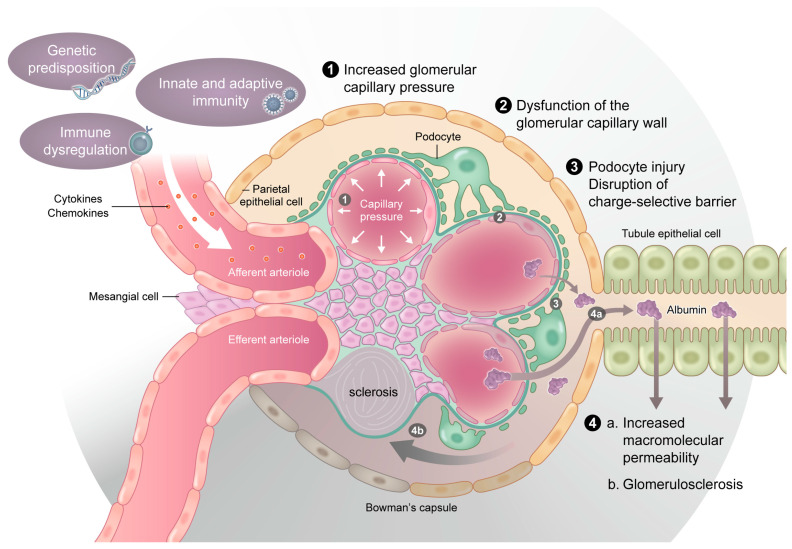
Conceptual framework illustrating the pathophysiological links between rheumatoid arthritis (RA) and chronic kidney disease (CKD). This figure summarizes the major mechanisms contributing to renal injury in RA, encompassing immune-mediated pathways (such as chronic systemic inflammation and autoantibody- or immune-complex-mediated glomerular injury) as well as non-immunologic mechanisms, including endothelial dysfunction, hemodynamic stress, and cardiometabolic factors. The framework highlights the bidirectional interaction between RA disease activity and CKD progression, integrating inflammatory and hemodynamic processes within a unified conceptual model. In addition, proteinuria is depicted as an active pathogenic mediator, whereby excessive albumin uptake by tubular epithelial cells induces the release of extracellular vesicles, including exosomes, which amplify local inflammation and fibrotic responses within the tubulointerstitium.

Taken together, RA-associated renal injury reflects a complex interplay between immune-complex deposition, chronic inflammation, drug-related nephrotoxicity, and emerging molecular pathways that promote endothelial dysfunction and renal fibrosis. These mechanisms are summarized schematically in Figure 2 and provide a biological rationale for early inflammation control and integrated renoprotective strategies.

## 5. Treatment and Management

Managing RA in patients with coexisting CKD requires a dual approach: effective control of systemic inflammation to prevent further renal injury, and tailored adjustment of pharmacologic regimens to preserve kidney function and avoid drug-induced nephrotoxicity. Tight disease control has been shown to slow the decline in renal function in RA, supporting the treat-to-target strategy, even in patients with impaired kidney function [23].

### 5.1. Adjustment of Anti-Rheumatic Medications

Methotrexate (MTX), a cornerstone drug used in RA management, is primarily excreted renally. In patients with CKD, especially those with eGFR < 30 mL/min/1.73 m^2^, MTX accumulation can lead to serious toxicity, such as bone marrow suppression, necessitating dose reduction or discontinuation [36,37]. Leflunomide, which is hepatically metabolized, should be used with caution in patients with CKD, although its active metabolite can accumulate in advanced renal dysfunction [38]. Sulfasalazine and hydroxychloroquine have relatively favorable renal safety profiles and may be continued in mild to moderate CKD, although adjustments may be required in end-stage renal disease or dialysis [37,39,40,41].

Calcineurin inhibitors, such as tacrolimus, although used in some refractory RA cases, should generally be avoided in patients with progressive CKD because of their dose-dependent nephrotoxicity [37,42]. If renal impairment develops during treatment, alternative treatment agents should be considered. Glucocorticoids require careful tapering because prolonged use can exacerbate hypertension and diabetes, indirectly worsening renal outcomes [2]. The risk of infection with glucocorticoid therapy has also increased in patients with CKD.

### 5.2. Pain Management and NSAID Use

Nonsteroidal anti-inflammatory drugs (NSAIDs) are commonly used for pain control in RA; however, they pose a significant risk to patients with CKD. NSAIDs impair glomerular perfusion by inhibiting prostaglandin synthesis, potentially leading to acute kidney injury (AKI), particularly in the context of volume depletion or coadministration with renin–angiotensin system inhibitors. Chronic NSAID use can cause interstitial nephritis and accelerate the progression of CKD. Therefore, NSAIDs should be minimized or avoided when possible, and if used, they should be prescribed at the lowest effective dose for the shortest duration, with close monitoring of renal function, urine output, and blood pressure [17]. Acetaminophen and weak opioids may be considered as alternative analgesics, with dose adjustments based on renal clearance.

### 5.3. Renal Protective Strategies (Clinical Implementation)

A risk-based CKD care plan aligned with KDIGO 2024 guidelines should be incorporated into routine RA management. First, we recommend at least annual eGFR and uACR screening (more frequent screening is required in patients with active disease, pre-existing CKD, or nephrotoxin exposure) and stage by G1–G5 and A1–A3 to guide risk-based care [18,19]. Because low-range albuminuria already has prognostic value in RA, “high-normal” uACR should be considered, even when eGFR is preserved [15]. Blood pressure should be controlled according to the guideline targets using angiotensin-converting enzyme (ACE) inhibitors/angiotensin receptor blockers (ARBs) for albuminuric CKD unless contraindicated [18,19]. Analgesia should be NSAID-sparing. If NSAIDs are unavoidable, the lowest effective dose should be used for the shortest duration with renal monitoring [17].

Renal dosing, avoidance criteria, and recommended monitoring parameters (e.g., complete blood count and serum creatinine for methotrexate) for non-JAK anti-rheumatic agents are summarized in Table 1 (see Table 2 for Janus kinase (JAK) inhibitors).

When indicated, sodium-glucose cotransporter 2 (SGLT2) inhibitors can be considered for albuminuric CKD with adequate eGFR, regardless of diabetes status, to slow CKD progression and reduce cardio–renal events, following product-specific eGFR thresholds, and monitor for volume depletion and mycotic infections [18,43,44]. Glucagon-like peptide-1 (GLP-1) receptor agonists are appropriate for patients with type 2 diabetes/obesity and high cardiovascular risk, and may provide kidney benefits (e.g., slower albuminuria progression); they should be considered alongside SGLT2 inhibitors when appropriate [45,46]. Comprehensive multimorbidity management, including lipids, glycemia, weight, smoking cessation, and vaccination (e.g., zoster), is essential to mitigate infection and cardiovascular risk in patients with CKD and those undergoing immunomodulatory therapy [18,19]. Early rheumatology–nephrology co-management is recommended when patients reach CKD stage G3b–G5 and/or exhibit persistent albuminuria of category A2 or higher, as these thresholds identify individuals at substantially increased risk of progression and complications [18,19].

**Figure 3:**
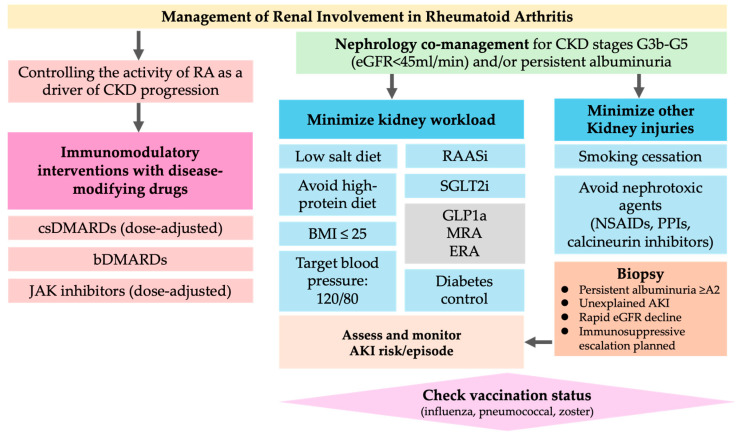
Management pathway for chronic kidney disease in patients with rheumatoid arthritis. This figure illustrates a stepwise management strategy for CKD in patients with RA, integrating immunologic disease control with renoprotective interventions. Initial risk stratification is based on estimated glomerular filtration rate and albuminuria category. Rheumatology–nephrology co-management is recommended for patients with CKD stage G3b–G5 and/or persistent albuminuria of category A2 or higher albuminuria (uACR ≥ 30 mg/g). Acute kidney injury risk factors, including NSAID exposure, dehydration, infection, and combined renin–angiotensin system blockade, should be actively assessed. Diagnostic kidney biopsy should be considered in cases of persistent albuminuria, unexplained AKI, or progressive renal dysfunction. Vaccination against influenza, pneumococcus, and herpes zoster is recommended prior to initiation of biologic or JAK inhibitor therapy whenever feasible. RA, rheumatoid arthritis; CKD, chronic kidney disease; csDMARDs, conventional synthetic disease-modifying anti-rheumatic drugs; bDMARDs, biologic DMARDs; JAK, Janus kinase; RAASi, renin–angiotensin–aldosterone system inhibitors; SGLT2i, sodium-glucose cotransporter 2 inhibitors; GLP-1a, glucagon-like peptide-1 receptor agonists; MRA, mineralocorticoid receptor antagonists; ERA, endothelin receptor antagonists; NSAIDs, nonsteroidal anti-inflammatory drugs; PPIs, proton pump inhibitors.

A pragmatic treatment pathway integrating risk staging, DMARD selection, and cotherapy is outlined in Figure 3.

### 5.4. Acute Kidney Injury, Kidney Biopsy, and Vaccination Strategies

Acute kidney injury represents an important but often underrecognized complication in patients with RA, particularly in the context of dehydration, infection, NSAID exposure, or combined renin–angiotensin system blockade [1,17]. Episodes of AKI may accelerate long-term CKD progression and adversely affect overall prognosis.

Regarding diagnostic evaluation, kidney biopsy should be actively considered in patients with RA who present with persistent albuminuria of category A2 or higher (uACR ≥ 30 mg/g), especially when the etiology is unclear, proteinuria is progressive, or immunosuppressive therapy is being escalated. Biopsy is also indicated in cases of unexplained AKI, rapidly progressive renal dysfunction, or suspected glomerulonephritis [18,19].

Because both immunosuppression and CKD heighten infection risk, vaccination plays a pivotal role in clinical management. Seasonal influenza, pneumococcal, and recombinant zoster vaccines should be administered according to international recommendations, ideally prior to initiation of biologic or JAK inhibitor therapy [47,48,49,50].

### 5.5. RA Treatment in Dialysis or Transplant Settings

Patients with RA undergoing dialysis require active disease management. As MTX and tacrolimus are contraindicated in end-stage kidney disease (ESKD), treatment regimens typically center on glucocorticoids and biologic agents. In patients with advanced CKD or those on dialysis, biologics do not require renal dose adjustment and demonstrate high treatment retention; vigilance for infection remains essential [51,52]. Balancing immunosuppressive therapy with disease control is challenging for kidney transplant recipients with RA. Escalating glucocorticoid doses should be avoided, if possible, and biologic DMARDs should be introduced to maintain disease remission while minimizing additional immunosuppression. Individualized treatment planning through rheumatology-nephrology collaboration is essential for optimizing both graft survival and RA control [37].

### 5.6. SGLT2 Inhibitors and GLP-1 Receptor Agonists: Mechanisms and Expected Benefits (Rationale & Evidence)

SGLT2 inhibitors reduce proximal tubular sodium–glucose reabsorption and restore tubuloglomerular feedback, thereby lowering intraglomerular pressure, reducing albuminuria by approximately 30%, and attenuating renal inflammation and fibrosis, which extend to diabetic and non-diabetic CKD [43]. GLP-1 receptor agonists improve weight, blood pressure, lipid levels, and inflammatory adipokine signaling, providing atherosclerotic cardiovascular risk reduction with kidney signals, including slower albuminuria progression [45,46].

Randomized trials have demonstrated disease-modifying kidney effects of SGLT2 inhibitors: DAPA-CKD and EMPA-KIDNEY reduced the composite endpoints of sustained eGFR decline, ESKD, or renal death, including non-diabetic CKD [43]. Large cardiovascular outcome trials of GLP-1 receptor agonists (e.g., LEADER and SUSTAIN-6) have shown reduced major adverse cardiovascular events (MACE) and albuminuria progression, with accumulating evidence of kidney protection [45,46].

Direct RA-CKD randomized trials with renal endpoints are lacking; however, given that the high cardiometabolic burden in RA and the prognostic impact of albuminuria, translating CKD guideline strategies to RA, is reasonable, although disease-specific trials are awaited [18,19,44,46]. Safety requires attention to volume depletion and mycotic infections with SGLT2 inhibitors and gastrointestinal/gallbladder adverse events with GLP-1 receptor agonists; choices should be individualized according to comorbidities and concurrent DMARDs [44,46]. Notably, biologic exposure is associated with a lower incidence of CKD than non-biologic regimens, suggesting that inflammation control and SGLT2i/GLP-1 RA co-therapies may be complementary in reducing residual cardiorenal risk [10,18,19,43,44,45,46].

## 6. Biologic and Targeted Therapies: Renal Implications of TNF Inhibitors, IL-6 Inhibitors, and JAK Inhibitors

The advent of biological disease-modifying anti-rheumatic drugs (bDMARDs) and targeted synthetic DMARDs (tsDMARDs) has revolutionized the treatment of rheumatoid arthritis (RA), offering improved joint outcomes and systemic benefits, including potential renoprotection in patients with RA. As chronic inflammation is a key driver of renal impairment in RA, suppression of pro-inflammatory cytokines may confer indirect but meaningful renal protection.

### 6.1. TNF-α Inhibitors

TNF inhibitors such as infliximab and etanercept have long been used in RA treatment. Their introduction significantly reduces systemic inflammation and acute-phase reactants, thereby contributing to a dramatic decline in the incidence of secondary AA amyloidosis [28]. A large retrospective cohort study using U.S. Veterans Affairs data showed that the use of biological agents, including TNF inhibitors, was associated with a lower incidence of CKD and a slower decline in the estimated glomerular filtration rate (eGFR) compared to non-biological therapies. Specifically, the annual rate of eGFR decline improved from −1.0 mL/min/1.73 m^2^/year to −0.4 mL/min/1.73 m^2^/year following the initiation of biologic treatment [10].

Contemporary comparative data from a multicenter Japanese cohort similarly suggested a renal benefit signal with TNF blockade: TNF inhibitors were linked to a lower CKD incidence than CTLA4-Ig (abatacept) (HR 0.67, 95% CI 0.46–0.97) [53].

Although TNF inhibitors are generally well tolerated in patients with CKD, rare cases of immune-mediated nephropathies, such as lupus-like nephritis and membranous nephropathy, have been reported, likely due to shifts in immune regulation. Causal inference is limited, but clinicians should monitor for new-onset proteinuria/hematuria during therapy [8,54,55].

### 6.2. IL-6 Inhibitors

IL-6 plays a central role in inflammation, SAA production, and subsequent amyloid deposition in AA amyloidosis. Tocilizumab, an anti-IL-6 receptor monoclonal antibody, has demonstrated excellent efficacy in controlling systemic inflammation and reducing SAA levels, making it particularly valuable in patients with RA and AA amyloidosis. In a single-center analysis from Heidelberg, patients with renal amyloidosis treated with tocilizumab had significantly better 5-year renal survival rates (71%) than those treated with TNF inhibitors (28%) [12]. These findings suggest superior renal preservation with IL-6 inhibition in the selected populations. Beyond amyloidosis, overall, biologic exposure is associated with a lower incidence of CKD and slower eGFR decline than non-biologic regimens in real-world data [10].

Tocilizumab is also advantageous in patients with CKD, where methotrexate coadministration is contraindicated, as it is highly effective as monotherapy [51,52]. Moreover, treatment persistence rates are higher with IL-6 inhibitors in patients with impaired renal function [52].

### 6.3. JAK Inhibitors

Janus kinase (JAK) inhibitors, such as tofacitinib, baricitinib, peficitinib, upadacitinib, and filgotinib, block the intracellular signaling of pro-inflammatory cytokines, including IL-6, and exert potent anti-inflammatory effects when administered orally. Their clinical utility has expanded substantially over the past decade; however, use in patients with chronic kidney disease (CKD) requires careful consideration because of differences in renal elimination, potential susceptibility to acute kidney injury (AKI), and an increased risk of infection [24].

#### 6.3.1. Pharmacokinetics and Renal Elimination

Pharmacokinetic profiles differ substantially among currently available JAK inhibitors. Baricitinib is predominantly eliminated through the kidneys, with approximately 70–75% of the administered dose excreted unchanged in the urine, necessitating strict dose reduction in patients with impaired renal function. In contrast, tofacitinib and upadacitinib are metabolized primarily by hepatic pathways, with renal clearance accounting for a smaller proportion of drug elimination, thereby allowing greater dosing flexibility in patients with mild to moderate CKD.

Despite these differences, dose adjustment is generally recommended for all JAK inhibitors when estimated glomerular filtration rate (eGFR) falls below 60 mL/min/1.73 m^2^, and most agents are contraindicated or not recommended in advanced CKD (eGFR < 30 mL/min/1.73 m^2^), depending on the specific compound and regulatory guidance.

#### 6.3.2. Renal Safety and Risk of Acute Kidney Injury

Evidence from large randomized controlled trials and post-marketing surveillance suggests that JAK inhibitors are not intrinsically nephrotoxic, and sustained declines in eGFR are uncommon under stable clinical conditions [53]. However, AKI may occur indirectly, particularly in vulnerable clinical settings. Episodes of infection, fever-related volume depletion, concomitant use of nonsteroidal anti-inflammatory drugs, or combined renin–angiotensin system blockade may predispose patients receiving JAK inhibitors to transient renal dysfunction.

Accordingly, baseline assessment and periodic monitoring of serum creatinine and eGFR are essential, especially during treatment initiation, dose escalation, and intercurrent illness.

#### 6.3.3. Infection Risk and Herpes Zoster in Patients with CKD

One of the most clinically relevant safety concerns associated with JAK inhibitors is the increased risk of herpes zoster, which consistently exceeds that observed with biologic DMARDs [50]. This risk is further amplified in patients with CKD, who exhibit baseline immune dysregulation and impaired antiviral immunity. Advanced age, renal impairment, and JAK inhibitor therapy act synergistically to increase the likelihood of severe or disseminated zoster infection.

For this reason, recombinant zoster vaccination should be strongly recommended prior to initiation of JAK inhibitor therapy, particularly in patients with CKD, in accordance with international vaccination guidelines [50].

#### 6.3.4. Comparative Renal Safety Among JAK Inhibitors

Agent-specific pharmacokinetic differences have important clinical implications for renal safety. Baricitinib requires the most stringent renal dose adjustment because of its high dependence on renal elimination, whereas upadacitinib demonstrates the least reliance on renal clearance among currently approved agents. These distinctions should be explicitly considered when selecting therapy for patients with CKD.

At present, no randomized controlled trials have directly compared renal outcomes among different JAK inhibitors in CKD-stratified RA populations. Available data are derived largely from post hoc analyses and observational studies, underscoring the need for cautious interpretation.

#### 6.3.5. Integration with CKD-Specific Management

In patients with RA and CKD, JAK inhibitors should generally be positioned after optimization of conventional synthetic and biologic DMARD therapy. Treatment selection should incorporate individualized dose adjustment based on eGFR, careful avoidance of concurrent nephrotoxic exposures, and close collaboration with nephrologists in patients with CKD stage G3b or higher. The dose-adjustment principles summarized in Table 2 provide a practical framework for daily clinical practice.

#### 6.3.6. Evidence Gaps and Future Perspectives

Despite the widespread use of JAK inhibitors, robust evidence regarding their long-term renal safety or potential renoprotective effects remains limited. Most pivotal trials excluded patients with advanced CKD and did not incorporate renal endpoints, such as albuminuria progression or AKI incidence, as primary outcomes. Future prospective RA trials incorporating CKD-specific endpoints and stratified analyses by renal function are urgently needed to inform optimal use of JAK inhibitors in this high-risk population.

The renal dose adjustments for JAK inhibitors are summarized in Table 2.

## 7. Prognosis and Outcomes

### 7.1. eGFR Trajectories and Predictors

Higher RA disease activity is consistently associated with faster eGFR decline, underscoring the renoprotective value of tight inflammatory control [23]. Traditional determinants, such as age, hypertension, diabetes, and established CVD, remain salient, but inflammatory biomarkers (e.g., CRP) and albuminuria provide incremental prognostic information in RA [5,15]. Longitudinal data indicate that a meaningful minority of patients with RA transition from preserved function to CKD over time; for example, eGFR < 60 accrued to ~15% at 10 years and ~25% at 20 years in a classic cohort [13].

### 7.2. Progression to End Stage Kidney Disease (ESKD)

RA increases the risk of kidney failure at the population level. In a nationwide Korean cohort of >930,000 individuals, the incidence of ESKD was 0.81 vs. 0.37 per 1000 person-years in patients with RA versus non-RA, yielding an adjusted HR of 2.10 (95% CI 1.90–2.31) over 7.6 years; thus, the absolute annual risk was low (~0.08%), but the relative risk was roughly doubled [56]. The excess appears more pronounced in younger patients with RA and those with fewer baseline comorbidities, implying that inflammatory mechanisms rather than competing risks dominate in some subgroups [56]. Secondary AA amyloidosis historically accounts for a share of kidney failure in RA; with biologic therapies, its incidence and renal progression have declined, but vigilance remains warranted in persistently inflamed diseases [20].

### 7.3. Cardiovascular Events and Mortality in RA with CKD

There is a vascular risk of CKD compounds in RA. In a Taiwanese nationwide RA cohort, the presence of CKD was associated with incident ischemic heart disease HR 1.57 (95% CI 1.38–1.79) and stroke HR 1.24 (1.06–1.43) versus RA without CKD [57]. In a Japanese RA cohort, CKD was a predictor of all-cause mortality (HR 1.64 [1.05–2.57]), which increased to HR 4.76 (2.24–9.51) in very high-risk CKD [2]. These data emphasize the need for integrated cardio–renal risk reduction (blood pressure, lipids, glycemia, and smoking cessation) along with inflammation control [4].

### 7.4. Albuminuria as an Early Prognostic Signal

In patients with RA, albuminuria poses a risk even at low levels. In the NHANES 1999–2018, the uACR–mortality relationship displayed an L-shaped threshold near 6 mg/g; above this level, mortality rose steadily [15]. Additional cohorts have reported approximately 1.5-fold higher mortality with microalbuminuria (30–300 mg/g) [16]. Clinically, routine uACR testing complements eGFR, improves stratification, and can trigger early renoprotective measures.

### 7.5. Clinical Take-Home Themes

Several themes emerged from these data. First, higher RA disease activity is consistently associated with a steeper decline in kidney function, underscoring the importance of inflammation control as a renoprotective strategy [23]. Second, albuminuria carries prognostic weight even at low ranges. RA cohorts show an L-shaped association between uACR and mortality, with a threshold of approximately 6 mg/g, and microalbuminuria (30–300 mg/g) confers additional risk [15]. Third, although the absolute annual incidence of ESKD among patients with RA is low (~0.08% in contemporary nationwide data), the relative risk is approximately double compared to that in individuals without RA [56]. Fourth, the coexistence of CKD and RA amplifies downstream events, with higher rates of cardiovascular complications and all-cause mortality than in RA without CKD [2,57]. Finally, therapeutic decision-making should adopt KDIGO-aligned, cardio–renal-conscious care—routine eGFR and uACR monitoring, ACEi/ARB for albuminuric disease, and NSAID-sparing analgesia—while integrating co-therapies such as SGLT2 inhibitors or GLP-1 receptor agonists when indicated [18,19,44,46].

## 8. Future Directions and Unmet Needs

Despite growing recognition of the close bidirectional relationship between rheumatoid arthritis (RA) and chronic kidney disease (CKD), several critical knowledge gaps remain. To date, most randomized controlled trials of disease-modifying anti-rheumatic drugs (DMARDs) have systematically excluded patients with advanced CKD, and renal outcomes have rarely been incorporated as primary or key secondary endpoints. Consequently, evidence guiding treatment decisions in this high-risk population is largely extrapolated from non-CKD cohorts. Future RA trials should therefore prospectively integrate CKD-specific outcomes, including longitudinal changes in estimated glomerular filtration rate (eGFR) slope, albuminuria trajectories, and the incidence of acute kidney injury (AKI), in line with contemporary nephrology trial frameworks.

Second, CKD-stratified trial designs are urgently needed to clarify the comparative renal safety and potential renoprotective effects of biologic and targeted synthetic DMARDs across different stages of kidney dysfunction. Observational studies suggest heterogeneity in renal risk profiles among tumor necrosis factor inhibitors, IL-6 inhibitors, and Janus kinase inhibitors, but robust head-to-head comparisons with renal endpoints are lacking. Stratification by CKD stage would allow more precise and individualized treatment selection in vulnerable populations with RA.

Third, emerging multi-omics technologies, including single-cell transcriptomics, spatial transcriptomics, and proteomics, provide unprecedented opportunities to dissect the cellular and molecular crosstalk between synovial inflammation and renal parenchymal injury. Recent applications of spatially resolved transcriptomic approaches in kidney disease have revealed distinct inflammatory and fibrotic niches that cannot be captured by bulk analyses. Integration of these advanced technologies with well-phenotyped RA cohorts may enable the identification of novel biomarkers and therapeutic targets specific to RA-associated CKD, thereby facilitating translational advances from bench to bedside.

Finally, interventional strategies explicitly targeting cardio–reno–metabolic risk warrant focused investigation in RA-specific CKD populations. Large cardiovascular and renal outcome trials have demonstrated that sodium–glucose cotransporter 2 inhibitors and glucagon-like peptide-1 receptor agonists confer substantial renoprotective and cardioprotective benefits in patients with CKD and diabetes or cardiovascular disease. However, patients with systemic inflammatory diseases such as RA have been underrepresented in these studies. Prospective trials evaluating these agents in RA-associated CKD may establish a new paradigm of integrated immunologic and renoprotective management.

## 9. Conclusions

Chronic kidney disease (CKD) is a frequent and clinically meaningful comorbidity in patients with rheumatoid arthritis, driven by the combined effects of traditional cardio–renal risk factors, persistent systemic inflammation, immune-mediated renal injury, and cumulative drug exposure. Even low-grade albuminuria below conventional microalbuminuria thresholds carries prognostic significance in RA, underscoring the importance of early detection.

Modern RA therapies have substantially reduced the burden of AA amyloidosis and may indirectly confer renal protection through tight inflammatory control. However, dose adjustment and vigilant safety monitoring remain mandatory for several DMARD classes, particularly JAK inhibitors and calcineurin inhibitors.

Routine surveillance of eGFR and uACR, risk-adapted pharmacologic adjustment, timely nephrology co-management, and individualized decisions regarding kidney biopsy are central to optimizing long-term outcomes.

Future RA research must move beyond joint-centered endpoints to incorporate renal and cardio–renal endpoints, thereby establishing truly integrated care strategies for this high-risk population.

## Figures and Tables

**Table 1:** Anti-rheumatic drugs in CKD (non-JAK): dosing/avoidance and renal considerations. Notes: Renal dosing needs and class-specific caveats are summarized; for JAK inhibitors, see Table 2. Abbreviations: AA amyloidosis, amyloid A amyloidosis; CBC, complete blood count; CKD, chronic kidney disease; eGFR, estimated glomerular filtration rate; NSAIDs, nonsteroidal anti-inflammatory drugs; TNF, tumor necrosis factor.

Drug	Renal Adjustment	Use in Advanced CKD (eGFR < 30)	Monitoring Parameters	Key Comments
**Methotrexate**	Reduce dose or extend interval at eGFR 30–59	Avoid	CBC; serum creatinine	Accumulation risk; myelosuppression
**Leflunomide**	No adjustment usually required	Use with caution	Liver enzymes; CBC; renal function	Active metabolite long half-life; limited CKD data
**Sulfasalazine**	No adjustment in mild–moderate CKD	Use with caution	CBC; renal function	Rare interstitial nephritis
**Hydroxychloroquine**	No adjustment usually required	Use with caution	Renal function; ophthalmologic exam	Partial renal clearance; retinal toxicity
**Glucocorticoids (low–moderate dose)**	No adjustment required	Can be used	Blood pressure; glucose; infection	Long-term metabolic and CV risk
**NSAIDs**	Avoid or minimize use	Avoid	Serum creatinine; electrolytes	AKI risk; CKD progression
**Tacrolimus**	Dose adjustment required	Avoid	Serum creatinine; trough level	Dose-dependent nephrotoxicity
**TNF inhibitors**	No adjustment required	Can be used	Renal function	No intrinsic nephrotoxicity
**IL-6 inhibitors**	No adjustment required	Can be used	Renal function; CRP	Beneficial for AA amyloidosis
**Abatacept**	No adjustment required	Can be used	Renal function	Favorable renal safety profile

**Table 2:** JAK inhibitors: suggested dosing based on renal function in RA. Notes: Dose adjustment recommendations are based on eGFR and refer to adult patients with RA. In patients with advanced CKD, particularly eGFR < 30 mL/min/1.73 m^2^, the use of JAK inhibitors should be approached with caution or avoided, depending on the specific agent, due to limited clinical data and altered pharmacokinetics. Baricitinib requires the most stringent renal dose adjustment because of predominant renal elimination, whereas upadacitinib and tofacitinib rely mainly on hepatic metabolism with less renal clearance. Peficitinib is primarily metabolized hepatically and may be used without dose adjustment in mild to moderate CKD; however, evidence in advanced CKD remains limited. Abbreviations: AKI, acute kidney injury; CKD, chronic kidney disease; eGFR, estimated glomerular filtration rate; JAK, Janus kinase; NSAID, nonsteroidal anti-inflammatory drug; RAAS, renin–angiotensin–aldosterone system.

JAK Inhibitor	Renal Elimination	Dose Adjustment in CKD	Use in Advanced CKD (eGFR < 30)	Renal Safety Considerations
**Baricitinib**	High (~70–75%)	Reduce dose at eGFR 30–59	Not recommended	AKI risk with dehydration, NSAIDs, RAAS blockade
**Tofacitinib**	Moderate (~30%)	Reduce dose at eGFR < 60	Not recommended	Stable eGFR in trials; monitor during illness
**Upadacitinib**	Low (<20%)	No adjustment in mild–moderate CKD	Avoid (limited data)	Least renal dependence; infection-related AKI risk
**Filgotinib**	Moderate (active metabolite)	Reduce dose at eGFR < 60	Not recommended	Limited CKD data; avoid advanced CKD
**Peficitinib**	Minimal (<15%)	No adjustment in mild–moderate CKD	Use with caution/avoid	Primarily hepatic metabolism; minimal renal exposure

## Data Availability

No new data were created or analyzed in this study.
